# Monitoring the quality of SARS‐CoV‐2 virus detection in molecular diagnostic laboratories in the Eastern Mediterranean Region during the COVID‐19 pandemic

**DOI:** 10.1111/irv.13217

**Published:** 2023-11-17

**Authors:** Luke W. Meredith, Mustafa Aboualy, Rachel Ochola, Mehmet Ozel, Abdinasir Abubakar, Amal Barakat

**Affiliations:** ^1^ Infectious Hazard Management, Department of Health Emergency World Health Organization, Eastern Mediterranean Regional Office Cairo Egypt

**Keywords:** COVID‐19, EMRO, laboratory, quality, SARS‐CoV‐2, surveillance

## Abstract

**Introduction:**

The COVID‐19 pandemic placed unprecedented stress on laboratories in the Eastern Mediterranean Region. Building on existing capacity for influenza diagnostics, countries introduced COVID‐19 diagnostic support to ~100% regional coverage. A key challenge during the expansion was maintaining quality testing in laboratories, ensuring that correct results were shared with medical facilities.

**Methods:**

WHO organized two rounds of independently monitored severe acute respiratory syndrome coronavirus‐2 (SARS‐CoV‐2) external quality assurance programs (EQAP). The Public Health Laboratory (PHL) division of WHO supplied external quality assurance (EQA) panels, from the Royal College of Pathologists of Australasia Quality Assurance Programme (RCPAQAP) Australia to laboratories not enrolled in recurring Global Influenza Surveillance and Response System (GISRS) quality assurance programs, in which national influenza centers routinely participate.

**Results:**

Fifteen and 14 countries participated in PHL/EQAP for SARS‐CoV‐2 between 2020 and 2022. Concordance was consistent between rounds, reaching 96.4% and 89.9%. A separate assessment of GISRS/EQAP to national‐level laboratories identified high levels of response and concordance for SARS‐CoV‐2 (100% response, 93% concordance), which was reduced for influenza (50% response rate, 80% concordance), reflecting the challenge of prioritizing pathogens during outbreaks.

**Conclusion:**

The proliferation of laboratories in response to COVID‐19 was a success story from the pandemic. However, monitoring the quality of laboratories was challenging via existing EQAP. The addition of PHL/EQAP provided a mechanism to monitor performance of laboratories that were not designated as national influenza centers. While a high proportion of laboratories attained good results, continual emphasis on quality and enrollment in EQAP is key to ensuring sustainability of laboratory testing in future.

## 1 INTRODUCTION

The World Health Organization Regional Office for the Eastern Mediterranean (WHO/EMRO) comprises the occupied Palestinian territory and 21 member states: Afghanistan, Bahrain, Djibouti, Egypt, Islamic Republic of Iran, Iraq, Jordan, Kuwait, Lebanon, Libya, Morocco, Oman, Pakistan, Qatar, Saudi Arabia, Somalia, Sudan, Syrian Arab Republic, Tunisia, United Arab Emirates, and Yemen.^1^ This region has diverse cultures, socio‐economic conditions, and demographic characteristics, meaning the provision of health and other services in the region can be challenging.^2^ Acute and protracted humanitarian emergencies, poverty, political instability, and fragile health systems require constant support and monitoring from national, regional, and international stakeholders to ensure health care is available to those who need it most.^3^

The COVID‐19 pandemic placed unprecedented pressure on public health systems in the Eastern Mediterranean Region (EMR), resulting in surges in the number of patients requiring treatment or support and the number of samples needing diagnostic testing in national or sub‐national laboratories. Accurate and early detection of severe acute respiratory syndrome coronavirus‐2 (SARS‐CoV‐2) utilizes real‐time reverse transcription polymerase chain reaction (rtRT‐PCR) as the most reliable method for diagnosing COVID‐19.^4^ Molecular testing requires technical skills and equipment that are often heavily utilized for multiple diseases, particularly true in the EMR, where outbreaks are frequent and widespread. As of March 2023, there were 41 active outbreaks^5^ in the region, each requiring dedicated laboratory testing and support.

Several countries were able to capitalize on existing laboratory infrastructure in national reference laboratories and national influenza centers (NICs). These facilities provide high‐quality and high‐throughput diagnostic and sequencing support for influenza and respiratory pathogens as part of the Global Influenza Surveillance and Response System (GISRS) and were rapidly able to support SARS‐CoV‐2 molecular testing.^2^ However, the volume of samples and the geographical distribution of COVID‐19 meant that capacity needed to be expanded beyond central locations. National authorities worked with WHO and other stakeholders to implement testing at various sub‐national laboratories, providing private, academic, institutional, or localized testing across their countries. Training and reagents were provided, resulting in a proliferation of laboratory testing capacity, allowing for near saturation coverage of the populations in the region, and improving response times to cases as they occurred.

As per WHO, a SARS‐CoV‐2 molecular testing assay with limit of detection as low as 100 copies of viral genomic material per each milliliter of any respiratory tract clinical specimen type, with over 95% sensitivity and 98% specificity, is acceptable to be used in SARS‐CoV‐2 diagnostics.^6^ The high sensitivity of testing means that laboratory workflows must be carefully monitored and maintained to ensure that false positive or negative results do not occur due to contamination, reagent mismatching, or other challenges which can impact the overall quality of the assays used. External quality assurance programs (EQAP) are a mechanism that provides confidence to all levels of the population that the results being obtained in a laboratory are correct. EQAP operate by providing samples of known provenance to participating laboratories. The laboratory then test samples using their existing protocols, and provide the results back to the EQAP. The EQAP then assess and compile the results on local, national, and international levels, to assure laboratories are attaining the same results for the same samples.

The NICs are enrolled in the GISRS quality assurance program (GISRS/EQAP), which has been running for many years and provides annual assessment of laboratory performance.^7^ This is a global network focusing on influenza, ensuring countries respond rapidly to new strains or variants of influenza as they emerge. The first SARS‐CoV‐2 external quality assurance was in April 2020, through the GISRS/EQAP but was only for GISRS network, that is, national level. As many countries expanded in SARS‐CoV‐2 testing through sub‐national laboratories, the existing breadth and scope of GISRS/EQAP network did not have the capacity to expand and integrate new COVID‐19 testing laboratories into the existing EQAP.

To fill the gap, Public Health Laboratory (PHL) Strengthening Unit in the Lyon Office of the World Health Emergencies Programme (WHE) of WHO headquarters organized a global laboratory proficiency testing program designed to include sub‐national laboratories (PHL/EQAP). Panels consisting of five specimens (2020/21) and six specimens (2022) were prepared and distributed, and participants were asked to test and report their results back to WHO. Participants were also given the opportunity to report multiple sets of results if they used several platforms for testing SARS‐CoV‐2 to ensure that as broad a panel of laboratory tests were assessed as possible. WHO/EMRO facilitated laboratory participation in the region to ensure that the results from member states were consistent with the global standards.

## 2 METHODS

### 2.1 Design and distribution of PHL/EQAP panels

The PHL/EQAP panel consisted of five (2020/21) or six samples (2022), designed and provided by the Royal College of Pathologists of Australasia Quality Assurance Programme (RCPAQAP). Inactivated tissue culture SARS‐CoV‐2 viruses were used as the source of the positive control specimens. Sterility testing for inactivated positive specimens was confirmed by non‐propagative in vitro cell line passages. Sample composition is shown in Table 1.

**TABLE 1:** Panel design for the two rounds of WHO SARS‐CoV‐2 PHL/EQAP.

EQA sample	Round No.	Estimated genome equivalents (GE)/mL	Virus	Expected results
WHO‐SC‐20‐01	First	4.5 × 10^4^	COVID‐19/SARS‐CoV‐2/Vic/1/2020	Positive
WHO‐SC‐20‐02	First	2.3 × 10^6^	COVID‐19/SARS‐CoV‐2/Vic/1/2020	Positive
WHO‐SC‐20‐03	First	3.5 × 10^6^	HCoV‐OC43	Negative
WHO‐SC‐20‐04	First	N/A	Diluted MDCK cells—negative sample	Negative
WHO‐SC‐20‐05	First	4.5 × 10^3^	COVID‐19/SARS‐CoV‐2/Vic/1/2020	Positive
WHO‐SC‐22‐01	Second	2 × 10^7^	COVID‐19/SARS‐CoV‐2/VIC/35864 (BA.2 Omicron)	Positive
WHO‐SC‐22‐02	Second	1 × 10^7^	HCoV‐229E	Negative
WHO‐SC‐22‐03	Second	3 × 10^7^	COVID‐19/SARS‐CoV‐2/VIC/18440 (Delta)	Positive
WHO‐SC‐22‐04	Second	3 × 10^4^	COVID‐19/SARS‐CoV‐2/VIC/18440 (Delta)	Positive
WHO‐SC‐22‐05	Second	N/A	Diluted MDCK cells—negative sample	Negative
WHO‐SC‐22‐06	Second	2 × 10^4^	COVID‐19/SARS‐CoV‐2/VIC/35864 (BA.2 Omicron)	Positive

*Note*: Internal control was not included in the design. Laboratories could choose to add this as per their operating protocols.

Samples did not include an internal control (human RNAse P gene) commonly used as extraction control in molecular diagnostic laboratories; participants could choose to add this or not, but it was not considered in the final analysis. Panel composition, sterility, concentration of required genome equivalent (GE) per milliliter, and packaging were managed by RCPAQAP. Panels were tested to assure integrity, stability, and sterility before they were cleared for distribution. Panels were shipped at room temperature to each country to a central point in each of the participating country, where the recipient country was responsible for internal distribution to further participating sub‐national laboratories.

Samples were required to go through the standard laboratory process, from extraction through to rtRT‐PCR and data analysis, and results supplied to the WHO PHL program for analysis.

### 2.2 Assessment and scoring criteria

Results were reported as positive or negative for consideration in the assessment. Positive, negative, inconclusive, or invalid results can all be obtained from laboratories, depending on the reporting algorithm for the diagnostic kit being used for SARS‐CoV‐2 and the cycle thresholds for reporting positive or negative results. For this PHL/EQAP, the focus was to assess correct or incorrect sample detection, meaning invalid results were not assessed, as they do not clearly define what was in the sample.

In the first round, concordance was attained if positive and negative results were correctly identified. Results reported as not tested or invalid were not scored or assessed. A discordant score was attained if the positive or negative was incorrectly reported, or if the result is reported as inconclusive.

In the second round, global scores were calculated using a similar approach, except that inconclusive results in round two were considered as “not tested” and subsequently not scored as a “discordant” result. In this manuscript, results were assessed following the protocol from the first round; inconclusive results were considered as discordant result.

### 2.3 Equipment and laboratory survey

A survey was included in the sample distribution to assess kit and protocol usage across the region and ask participants to identify key factors that will impact the performance during the first cycle of PHL/EQAP. A drop‐down list of the most common PCR machines, reagents, and kits was included, but an option was also provided for manual data entry. The results were compiled and assessed to gather an understanding of the factors impacting the results of the PHL/EQAP.

## 3 RESULTS

### 3.1 Regional performance of the two rounds of PHL/EQAP

**FIGURE 1:**
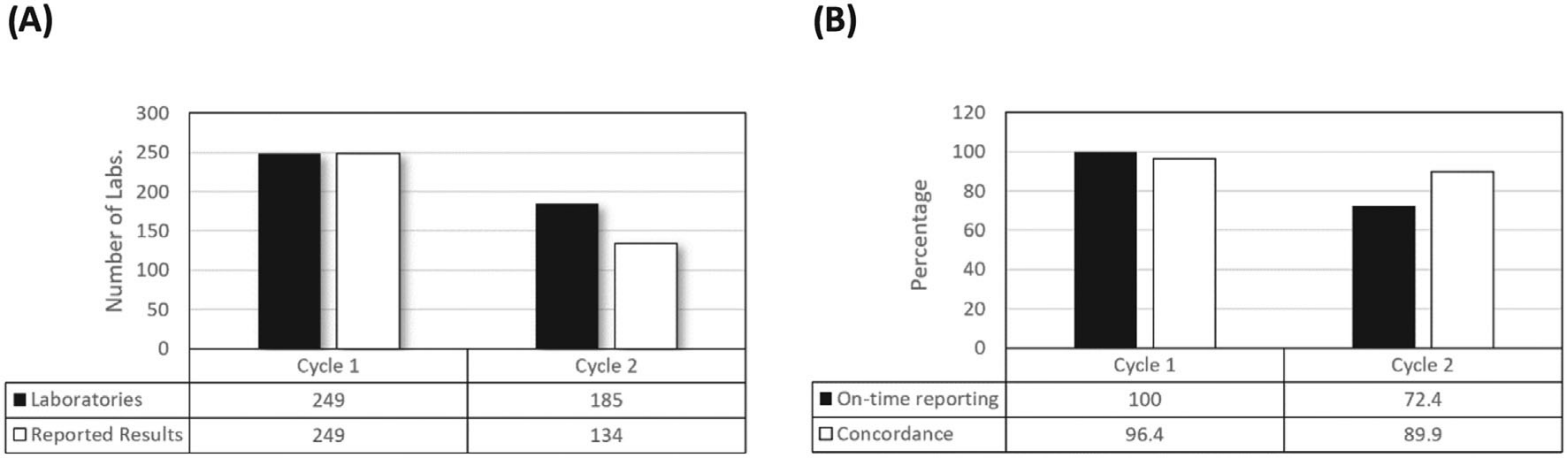
Participation and results of the 2020–2022 SARS‐CoV‐2 EQA results. (A) Two hundred forty‐nine laboratories from 15 countries participated in the 2020/21 EQA cycle, with 100% returning results for assessment. By comparison, 185 laboratories participated in the second cycle of testing, with 51 laboratories (27.6%) not returning results within the testing timeline. (B) Concordance results for the first cycle of testing (2020/21) showed 96.4% (range 86.4% to 100.0%) of samples were correctly identified by sub‐national facilities across the region, compared with 89.9% (range 66.7% to 100.0%) in Cycle 2 (2022).

The WHO PHL program has run two cycles of EQA testing since 2020, with a cycle covering 2020/21 and a second cycle in 2022. In the first cycle, 249 laboratories were enrolled in the program, with 100% of laboratories returning results within the required timeframe. By comparison, in the second cycle, the participation rate showed 72.4% on‐time result submission; out of the 185 enrolled laboratories, 134 submitted results, and 51 laboratories did not submit back any result (Figure 1).

An analysis of the concordance results showed that overall, the region attained a positive concordance 96.4% in Cycle 1, compared with 89.9% in Cycle 2. Following the same scoring method, and analyzing only the submitted results of either concordant or discordant, excluding the “not tested” and “invalid” submissions, the global concordance average is 97.3% (12,042/12,374) for Cycle 1 and 93.4% (15,572/16,668) for Cycle 2—noting that the number of discordant result in the global scoring for Cycle 2 does not include inconclusive submissions and was excluded from the percentage along with the not tested and invalid submissions.

A deeper analysis of the regional scores, compiling results of 249 participating laboratories in Cycle 1—representing 15 EMR countries (Afghanistan, Bahrain, Egypt, Islamic Republic of Iran, Jordan, Lebanon, Libya, Morocco, Oman, Pakistan, Qatar, Somalia, Sudan, Tunisia, and Yemen)—showed a total of 1245 submitted results, out of which 81 submissions (6.5%) were submitted as “not tested” or “invalid test” and subsequently were not analyzed. Submitted data for analysis were 1164 submissions for the five samples. Total correct submissions were 1122 (96.4%) and 42 incorrect and inconclusive results (3.6%). The 42 discordant results constitute 19 inconclusive, 15 false negatives, and eight false positives. Result of both cycles is shown in Table 2.

**TABLE 2:** Results of aggregated regional performance of each specimen for the SARS‐CoV‐2 PHL/EQAP showing numbers and percentages of concordant and discordant scores for the two rounds.

Sample #	EQAP round	Total submissions	Samples reported as invalid or not tested	Total submissions to be analyzed	Total correct results	False positive results	False negative results	Inconclusive results	Total incorrect results
WHO‐SC‐20‐01	1	249	1	248	246 (246/248 = 99.2%)	N/A	1	1	2 (2/248 = 0.8%)
WHO‐SC‐20‐02	1	249	5	244	243 (243/244 = 99.6%)	N/A	1	0	1 (1/244 = 0.4%)
WHO‐SC‐20‐03	1	249	6	243	234 (234/243 = 96.3%)	6	N/A	3	9 (9/243 = 3.7%)
WHO‐SC‐20‐04	1	249	64	185	175 (175/185 = 94.6%)	2	N/A	8	10 (10/185 = 5.4%)
WHO‐SC‐20‐05	1	249	5	244	224 (224/244 = 91.8%)	N/A	13	7	20 (20/244 = 8.2%)
Total per panel in Round 1		1245	81	1164	1122 (1122/1164 = 96.4%)	8 (0.7%)	15 (1.3%)	19 (1.6%)	42 (42/1164 = 3.6%)
WHO‐SC‐22‐01	2	134	1	133	129 (129/133 = 96.9%)	N/A	4	0	4 (4/133 = 3%)
WHO‐SC‐22‐02	2	134	4	130	121 (121/130 = 93.1%)	6	N/A	3	9 (9/130 = 6.9%)
WHO‐SC‐22‐03	2	134	1	133	128 (128/133 = 96.2%)	N/A	4	1	5 (5/133 = 3.8%)
WHO‐SC‐22‐04	2	134	3	131	108 (108/131 = 82.4%)	N/A	18	5	23 (23/131 = 17.6%)
WHO‐SC‐22‐05	2	134	7	127	118 (118/127 = 92.9%)	7	N/A	2	9 (9/127 = 7.1%)
WHO‐SC‐22‐06	2	134	5	129	100 (100/129 = 77.5%)	N/A	24	5	29 (29/129 = 22.5%)
Total per panel in Round 2		804	21	783	704 (704/783 = 89.9%)	13 (1.7%)	50 (6.4%)	16 (2%)	79 (79/783 = 10.1%)

Of those 249 laboratories participated in this cycle, 58 labs tested the panels through two different platforms (primary and secondary platforms) and submitted different data sets, representing in total 307 data set submissions, from the 249 laboratories. In the context of this manuscript, platform refers to both the PCR machines and the PCR assays being used, as both the type of machine and assay can have an impact on the sensitivity of detection. It is worth noting that the results shown in Table 2 for the first cycle focused on the primary platforms for all countries and does not reflect results of the second platform. Comparative results of the primary and secondary platforms used by the 58 laboratories are shown separately in Table 3.

**TABLE 3:** Results of the two platforms used by 58 laboratories in Round 1 of the PHL/EQAP representing 11 countries (Afghanistan, Bahrain, Islamic Republic of Iran, Jordan, Lebanon, Libya, Oman, Pakistan, Qatar, Somalia, and Tunisia).

Parameter	Platform 1	Platform 2
Total submissions	290	290
Samples reported as invalid or not tested	12	14
Total submissions to be analyzed	278	276
Total correct results	264 (95%)	258 (93.5%)
False positive results	2 (0.7%)	3 (1.1%)
False negative results	6 (2.2%)	8 (2.9%)
Inconclusive results	6 (2.2%)	7 (2.5%)
Total incorrect results	14 (5%)	18 (6.5%)

For the second cycle, aggregated results of the 134 participating laboratories representing 14 EMR countries (Bahrain, Egypt, Iraq, Islamic Republic of Iran, Jordan, Libya, Oman, Pakistan, Qatar, Saudi Arabia, Somalia, Syrian Arab Republic, United Arab Emirates, and Yemen) showed a total of 804 submitted results, out of which 21 submissions (2.6%) were submitted as not tested or invalid test and subsequently were not analyzed. Submitted data for analysis were 783 submissions for the six samples. Total correct submissions were 704 (704/783 = 89.9%) and 79 incorrect and inconclusive results (79/783 = 3.6%). The 79 discordant submissions comprise 50 false negatives, 16 inconclusive, and 13 false positive. In the second round, laboratories submitted data of single platform.

### 3.2 Comparative scores of the two platforms used by laboratories in PHL/EQAP first round

Concerning the 58 laboratories from 11 countries tested samples through two different platforms, the overall concordance of Platform 1 is 95% and 94% for Platform 2. Out of the 58 laboratories, results were identical between the two platforms in 54 laboratories (93%) whereas four laboratories (6.9%) from three countries reported different results for each platform. Results of concordant and discordant scores for the two platforms are shown in Table 3.

### 3.3 Results of equipment and reagent survey in PHL/EQAP Cycle 1

One of the key challenges faced globally was the availability of reagents and testing equipment to implement molecular testing for SARS‐CoV‐2. At the time of the pandemic declaration, testing kits were largely limited to the assay published by Corman et al.,^8^ provided by WHO through TIB Molbiol Syntheselabor GmbH (Berlin, Germany). However, in response to the pandemic and the need for additional testing capacity, commercial manufacturers produced hundreds of kits for use, which were distributed either directly or through national or international donations and collaborations.^9^, ^10^ The sensitivity and specificity of these assays can vary greatly depending on the targets and reagents included in the kit and whether they are validated on the PCR platforms being used.

**FIGURE 2:**
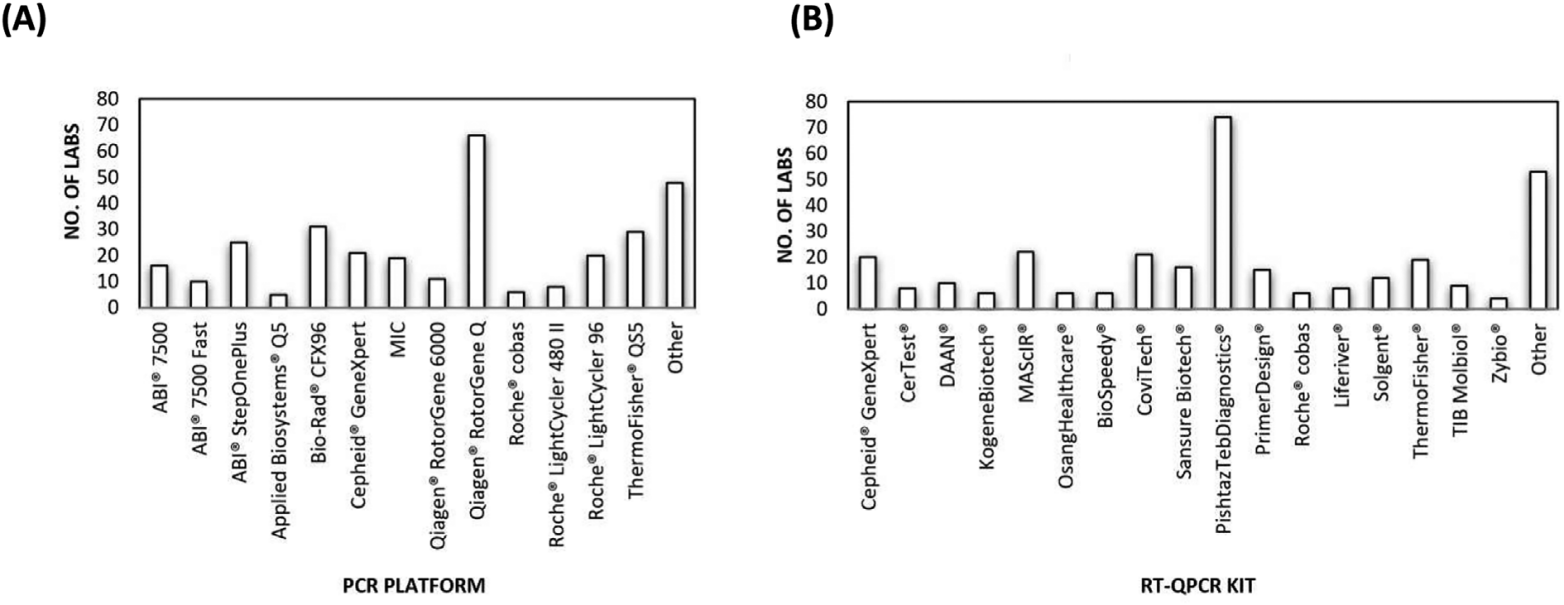
A diverse range of assays and equipment may be contributing to concordance results across the region. (A) An assessment of the PCR platforms across the region identified no fewer than 21 different PCR platforms being used across the region. (B) An assessment of the PCR kits being used identified 14 different assays being used, ranging from highly automated through to low throughput. The graphs illustrate the number of labs using each assay or kit (reflected as “No. of Labs” on the axis).

The diversity of kits used for the PHL/EQAP by sub‐national laboratories was assessed through a survey, requesting the laboratories identify the kits being used for extraction, as well as the PCR reagents, and the PCR machine being used for testing. The results identified that no fewer than 24 different PCR kits are being used across the region (Figure 2).

### 3.4 NICs faced challenges maintaining high levels of participation and performance in influenza EQA programs (GISRS/EQAP) as a result of increased focus on COVID‐19

The GISRS/EQAP was modified to include COVID‐19 in 2020, with NICs adding this to their annual quality assessments. This panel differed from the PHL/EQAP, so making a direct comparison between GISRS/EQAP and PHL/EQAP is not a component of this manuscript. However, it does provide an insight into the challenges of prioritizing mass testing of one pathogen over another in larger scale laboratories.

**FIGURE 3:**
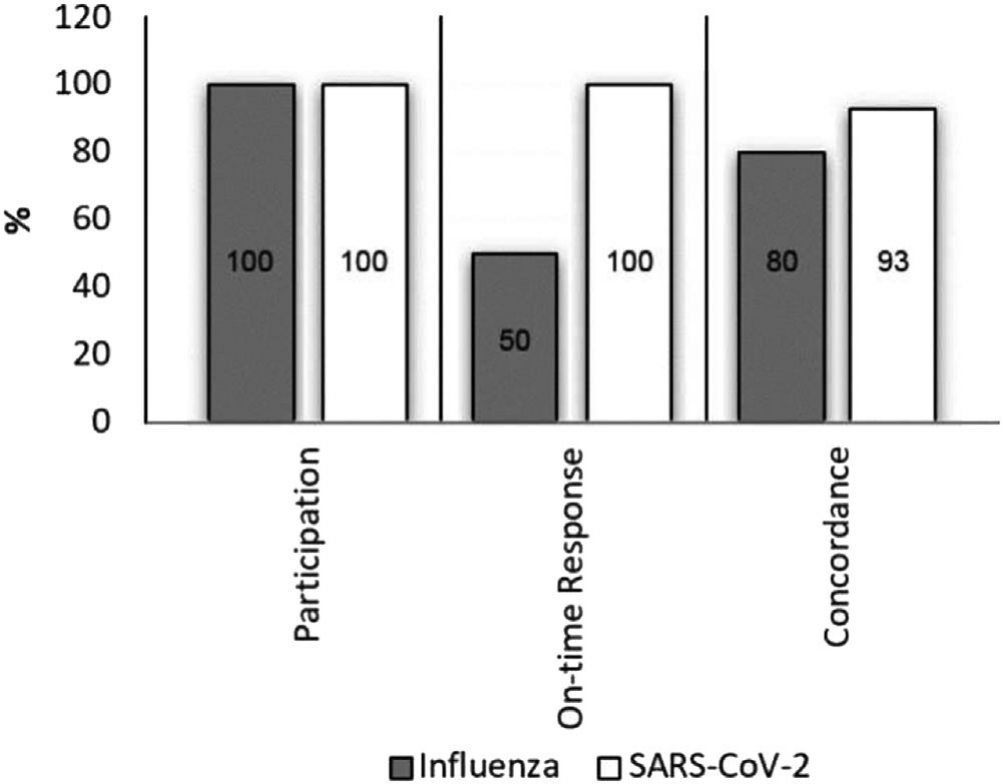
GISRS/EQA identifies a discordance between the quality of SARS‐CoV‐2 testing and influenza results. The results of the 2020/21 GISRS/EQAP were compiled and assessed for key performance indicators, including number of labs participating, response rates and time, and concordance with expected results.

Twenty countries in the EMR participated in the GISRS/EQAP. Of these, 28 NICs and laboratories testing for both SARS‐CoV‐2 and influenza A and B were included in the assessment. One hundred percent of laboratories participating provided results for both SARS‐CoV‐2 and influenza, but only 50% of the laboratories provided influenza results on time, compared with 100% of SARS‐CoV‐2 results. Similarly, concordance results showed that SARS‐CoV‐2 testing was 93% concordant with expected results, while influenza results were lower at 80% (Figure 3).

## 4 DISCUSSION

The expansion of laboratory testing across the EMR was one of the key success stories to emerge from the COVID‐19 pandemic.^2^ The ability of the NICs and National Public Health Laboratories to operationalize and scale up testing for SARS‐CoV‐2, capitalizing on the infrastructure developed through the GISRS network, is a step in the right direction for future pandemic responses. However, centralized testing has limitations, including logistical requirements to transport samples to the central locations, which are often non‐trivial, considering national circumstances. Limited logistical infrastructure, as well as available materials for maintaining a cold‐chain, can impact the quality of samples being transported to the national laboratories, impacting the quality of results that these facilities can offer.

One of the main challenges that many EMR countries are facing is the transport of specimens and reagents; regional participation rate in the PHL/EQAP rounds were affected by this limitation: In the first round, two countries globally could not receive the panels on time, one of the two countries was from the EMR, enrolling for 21 laboratories, while in the second round, three countries from the EMR (33.3%) out of the nine who globally could not receive the panels and participate, due logistical challenges, such as service interruptions by courier providers, import and export clearance issues and documentation concerns, noting that the nine countries are from four different WHO regions.

Additionally, EMR countries showed high dedication to submit results; overall aggregated regional participation percentage for the two rounds was 88.2% (249/249 = 100% Round 1 and 134/185 = 72.4% Round 2), while the global average was 82.4% (1809/2178 = 83.1% Round 1, 2934/3579 = 82% Round 2).

The change in the number of laboratories participating in the program is multifactorial. Logistical challenges with distributing kits across the geographical region were identified as a contributing factor. Funding challenges preventing laboratories from continuing to test for SARS‐CoV‐2 and changing national priorities were also identified as factors affecting the breadth of testing through the sub‐national laboratory networks, as well as affecting human resources, with staff being reallocated to alternative testing programs or laboratories.

The aggregated regional performance for each sample of the first and second cycles of the PHL/EQAP were compiled. The lowest concordance rate in the first round was reported as 91.8% correct for Sample Number 5 in the first cycle, with 8.2% false and inconclusive results (13 submissions of false negatives and seven inconclusive submissions). The lowest performance in the first cycle can be correlated to the low titer of the specimen; the lowest titer (Sample Number 5 with concentration of 4.5 × 10^3^ GE/mL) showed the lowest concordance response and the highest false negative submissions.

A similar result was also noticed in the second cycle, where the sample with the lowest titer (Sample Number 6 with a concentration of 2 × 10^4^ GE/mL) showed the lowest concordance rate with 77.5% correct results and with the highest number of submitted false negative (24 submissions, representing 18.6%).

Additionally, a key finding of this assessment is that as NIC redirected their efforts towards testing SARS‐CoV‐2, it directly impacted the quality of testing for influenza.^11^, ^12^ Those laboratories participating in the GISRS/EQAP showed strong performance in SARS‐CoV‐2 detection (93% concordance) but a lower EQA concordance result for influenza (80% concordance). This is to be expected, as constraints in equipment, human resources, and reagents will impact on the ability of an institution to run high‐throughput testing of a priority pathogen, such as SARS‐CoV‐2, without an expected impact on other testing programs.^13^, ^14^ This is also supported by the reduced response rate for the influenza EQA, which showed 50% of results reported on time, compared with 100% for SARS‐CoV‐2. This supports not only the case for expanding testing beyond central facilities but also the need for continued investment in capacity building in these facilities to ensure retention of skilled staff and continual improvement in quality testing of multiple pathogens.

This slight reduction in concordance can be attributed again to several factors. The EQA program was expanded to include additional samples, increasing pressure on the testing systems. However, it is important to note that the addition of a single sample should have minimal impact on the results. The decreased number of laboratories enrolled in the program could also impact the overall concordance results, while changes in human resources and testing platforms can directly affect testing results. Laboratories were independently asked to identify the key challenges they believed were impacting their results. Reagent availability, storage conditions, turnaround time, and human resources were identified as the key factors (data not shown).

The results shown here identified that sub‐national laboratories are highly competent, achieving comparable concordance to those identified through the GISRS/EQAP. However, a direct comparison between these two EQAP was avoided, due to the volume of laboratories testing in each program (28 in the GISRS/EQAP, testing for both SARS‐CoV‐2 and influenza compared with 309 in the PHL/EQAP testing only for SARS‐CoV‐2). However, they also identified a number of challenges that will impact the continued quality of testing in sub‐national facilities. The number of different tests being used across the region, and the differences in testing platforms and equipment, meant that interpretation of results in these laboratories will be highly variable.^15^ The PHL/EQAP and GISRS/EQAP results likely reflect the redirection of both technical and logistical capacity to SARS‐CoV‐2 in these facilities. Through the survey, factors affecting this were identified as machine and reagent availability, both at the extraction and PCR stage, as well as human resource availability, with the focus of training being SARS‐CoV‐2 for an extended period.

Decentralization of testing is a key to ensuring rapid response, turnaround times, and improved national and regional coverage during outbreaks.^16^, ^17^ Smaller labs servicing local catchment areas provide an invaluable resource to contribute to national diagnostic surveillance, whether private, public, academic, or cross‐sectoral, such as veterinary or environmental testing facilities. The expansion of this One Health approach to health, for everything ranging from research and discovery^18^, ^19^ to the diagnosis of zoonotic pathogens,^20^ is a key to ensuring that systems can operate together smoothly in the event of another pandemic occurring. The expansion of COVID‐19 testing from national laboratory facilities to sub‐national regions was a success story in the region, with capacity expanding from a limited number of national facilities to 200 or more sub‐national laboratories, which resulted in near 100% coverage of the region. However, while the expansion is to be encouraged, steps must be taken to ensure that laboratories provide the correct results, to avoid losing the trust built through national laboratory systems.

As presented in the Section 3, the sensitivity and specificity of an assay is directly linked to the extraction technique and the PCR platform being used for detection.^6^, ^9^ Certification marking in vitro diagnostics (CE‐IVD) in the European Union (EU) and Food and Drug Administration (FDA) requirements in the United States, for example, provide guidelines for the performance characteristics of an assay.^6^ These are provided in the instructions for use with an assay. However, in order to ensure that sub‐national facilities are attaining optimal results, it is critical that they be encouraged to assess the sensitivity of an assay with their own workflows, using established standards such as those provided by the National Institute for Biological Standards and Control (NIBSC), European Virus Archive‐Global (EVAG), US Centers for Disease Control and Prevention (CDC), or similar.^21^, ^22^, ^23^ This would be expected to improve concordance results in future and should be considered when implementing wider testing panels.

The sensitivity and specificity of so many tests is difficult to measure, but can directly impact whether a result is positive, negative, or inconclusive.^9^ Similarly, the study identified 26 different PCR platforms being used for testing and over 24 testing kits. It is important to note that the sensitivity and specificity of an assay is often measured on a single platform, and the instructions for use will reflect this. The performance characteristics of a PCR assay on a particular platform will have a direct impact on the correct assessment of a result and should be taken into consideration when decentralized testing is undertaken.^9^

Quality assurance is a key indicator of laboratory performance and contributes directly to the sustainability of diagnostic programs globally. EQAP such as those run by GISRS have ensured that results are comparable across regions. Commercial programs, such as Quality Control for Molecular Diagnostics Company (QCMD®), are available^24^ but are often cost‐prohibitive in developing countries, as well as having challenges with distribution through commercial channels. It is critical that national and international authorities continue to work together to ensure that these programs are available for enrollment and to encourage all laboratories to participate.

## 5 CONCLUSION

The PHL/EQAP implemented during the peak of the COVID‐19 pandemic contributed much in the continuous improvement process in regional laboratories. Member states were able to monitor the percentage of concordance and implement measures such as trainings and reviewed operating procedures specifically in laboratories with lower concordance scores.

Quality is a key aspect of the sustainable operation of laboratories both in EMR and globally. Continued investment in programs such as the WHO Laboratory EQA for COVID‐19 will build trust in laboratory systems and networks, allowing for improved coordination of responses for outbreaks in future. Support for these programs and continued encouragement for member states to enroll national and sub‐national laboratories into EQAP will remain a key focus of WHO/EMRO looking to the future.

## AUTHOR CONTRIBUTIONS

Abdinasir Abubakar, Amal Barakat, Rachel Ochola, and Mehmet Ozel conceptualized the paper. Luke W. Meredith drafted the manuscript with assistance from Mustafa Aboualy, Rachel Ochola, and Mehmet Ozel. All authors reviewed and contributed to subsequent drafts for important intellectual content and approved the final manuscript. The authors alone are responsible for the views expressed in this publication, and they do not necessarily represent the decisions or policies of WHO/EMRO.

## CONFLICT OF INTEREST STATEMENT

All authors are employed by WHO/EMRO.

## Data Availability

Data sharing is not applicable to this article as no new data were created or analyzed in this study.
